# Time to look beyond (and before) journal reporting requirements: a review of ethnicity reporting in UK clinical trial results publications in three high-impact journals

**DOI:** 10.1186/s13063-024-08596-7

**Published:** 2024-12-20

**Authors:** Mahwar Khanum, Shoba Dawson, Jhulia dos Santos, Huda Hajinur, Carmel Conefrey, Sangeetha Paramasivan

**Affiliations:** 1 Population Health Sciences, Bristol Medical School, University of Bristol, 39 Canynge Hall, Whatley Road, Bristol, BS8 2PS UK; 2 Caafi Health, Bristol, UK

Ethnic minority (EM) groups are consistently under-represented in health research, including in conditions that disproportionately affect them (e.g. cardiovascular diseases), with this paradox notably laid bare during the COVID-19 pandemic [1]. Reviews have extensively documented the lack of ethnicity reporting in clinical trial publications globally [2, 3].

We conducted a review to investigate the proportion of UK-based randomised controlled trial (RCT) results publications that reported on participants’ ethnicity in three high-impact factor medical journals—*The Lancet*, *New England Journal of Medicine* (NEJM) and *British Medical Journal* (BMJ)—from 1st January 2020 to 6th July 2022 (start date based on existing strong evidence of poor practice pre-pandemic). We searched the three journals’ websites for ‘trial’ in the title and included all UK-based RCTs (international RCTs were only included if at least 50% of the sites were from the UK). Data extraction focused on three key areas: was ethnicity data reported; if yes, what information was collected and how was it used (e.g. sub-group analysis); if no, was this discussed or acknowledged as a limitation.

Of the 367 records identified, we screened 118 full texts and included 68 articles (Lancet 49; NEJM 3; BMJ 16). More than half the studies reported some ethnicity data (56%; 38), but more than a third of these (37%; 14) did not provide a detailed ethnicity breakdown (i.e. they stated population numbers/proportion for the White group only, with all other ethnic groups combined). When detailed ethnicity data was provided, this was usually broken down by intervention group, but the categories and the depth of information varied widely (see Table 1 for examples of ethnicity data collected). Only one study provided a detailed breakdown of the White ethnic group (English/Welsh/Northern Irish/British, White Irish, Any other White background). Of the studies that collected some ethnicity data, half (50%; 19) did not use these in the analysis or mention the significance of this ethnicity data. Only over a quarter (29%; 11) used it in their analysis (where the primary outcome was provided by ethnicity) and about a fifth (21%; 8) used it to acknowledge the lack of ethnic diversity in their study in the Discussion section (but not always as a limitation). Finally, given the heightened focus on ethnicity since early in the pandemic, we expected studies that did not report ethnicity data (44%; 30) to acknowledge the limitation this poses or discuss ethnicity in some manner, but most did not (93%; 28). Two studies that did not report ethnicity data (7%) mentioned a lack of ethnic diversity in the study in their Discussion, but this was not acknowledged as a limitation.

**Table 1:** Ethnicity reporting in UK RCT results publications in three high-impact journals

Ethnicity reporting in three high-impact journals (*n* = 68)	*n* (%)
1. Studies reporting some ethnicity data	38 (56%)
1.1. What ethnicity data was captured?	
1.1a. Detailed ethnicity breakdown and by intervention group. E.g • White, Asian (Indian, Pakistani, Bangladeshi, Other), Black (Caribbean, African, Other), Mixed (Caribbean, African, Asian or Other) • White, (South or Southeast) Asian, Black, Mixed, Other • White, Asian, Mixed, Other • White, Asian, Black, Chinese, Mixed, Other, Prefer not to say	24 (63%)
1.1b. Insufficient detail in ethnicity breakdown (mostly by intervention group except in three studies). E.g • White and Other • White, Black/Asian and unknown	14 (37%)
1.2. How was the ethnicity data used?	
1.2a. In data analysis (e.g. primary outcome provided by ethnicity)	11 (29%)
1.2b. To note lack of ethnic diversity in study (with/without acknowledgement of this as a limitation and sometimes to add that this reflects the general population)	8 (21%)
1.2c. Not used/no further mention of ethnicity data	19 (50%)
2. Studies not reporting ethnicity data	30 (44%)
2.1. Mention of ethnicity in discussion (to note lack of ethnic diversity in study but not as a limitation)	2 (7%)
2.2. No mention anywhere in article (with one article noting provision of study leaflets in local languages during recruitment but no further detail or mention of ethnicity)	28 (93%)

*RCT* Randomised controlled trial

As researchers working within trials, we understand the challenges in ensuring ethnicity and socio-economic data are collected [4]. Some journals have introduced mandatory minimum reporting requirements for the representativeness of the study group, including on race or ethnic group [5, 6]. However, for tangible changes to redress the consistent under-reporting of ethnicity data in UK RCTs, we need more than journal reporting requirements for RCT publications, given they arrive at the end of the research cycle. There must be supportive mechanisms, driven by key players who engage with trialists earlier in the research pipeline and can effect change (e.g. funders), to enable trial teams to include ethnic minority participants as well as capture detailed ethnicity data. This means mandatory data collection requirements for trialists stipulated by funders (matched by appropriate resource allocation towards this), which are then reinforced by ethics committees and journals that publish protocols (e.g. *Trials*). Joined up, collaborative thinking across key stakeholders and players—from patients and public to health and community activists, researchers/trialists, clinicians, funders, ethics committees and journal editors—is required to ensure sustainable and impactful changes that improve the representativeness of our trial populations.

## Data Availability

All analysed data relevant to this study are included in the manuscript. The dataset on which this work is based consists of articles already available within the published literature.
